# Neuroimaging features of genetic syndromes associated with CNS overgrowth

**DOI:** 10.1007/s00247-022-05408-5

**Published:** 2022-10-08

**Authors:** Anthony R. Zamary, Mark D. Mamlouk

**Affiliations:** 1 Department of Radiology and Biomedical Imaging, University of California, San Francisco, San Francisco, CA USA; 2 Department of Radiology, The Permanente Medical Group, Kaiser Permanente Medical Center Santa Clara, 700 Lawrence Expy., Santa Clara, CA 95051 USA

**Keywords:** Central nervous system, Children, Hemimegalencephaly, Magnetic resonance imaging, Overgrowth syndromes, PIK3CA, Vascular malformations

## Abstract

Overgrowth syndromes can manifest with enlargement of the brain and other body parts and are associated with malignancy. Much of the current literature focuses on the imaging findings of the somatic overgrowth, while there is relatively little describing the overgrowth of the central nervous system. In this pictorial essay, we discuss common syndromes with central nervous system overgrowth, highlight key imaging features, and review the underlying genetics, including the PI3K-AKT-mTOR pathway as well as other syndromes from various genes.

## Introduction

Overgrowth syndromes comprise a heterogeneous group of disorders with excessive tissue development that can involve any body part, particularly the brain and extremities. Manifestations of overgrowth syndromes can range from minimal somatic overgrowth to major disfigurement and developmental delay. In addition, certain syndromes have a predisposition to cancer [1].

Historically, overgrowth syndromes were largely considered separate entities with variable pathogenesis, and the genetics were not well understood. This framework has changed with greater understanding, and we are now able to categorize overgrowth syndromes based on specific genes and gene classes [2]. For example, one of the most noteworthy gene pathways is the PI3K-AKT-mTOR pathway, whereby mutations in this pathway result in several overgrowth syndromes. Furthermore, knowledge of the genetics has led to targeted treatments for these syndromes.

Discussion of the radiologic findings of overgrowth syndromes has predominantly focused on features of the body, while less has been described within the brain and spine. Furthermore, much of the literature concerning the neuroimaging findings is piecemeal and focuses on a single syndrome, which can make it challenging to compare the different entities in routine clinical practice. The purpose of this pictorial review is to highlight the major syndromes with central nervous system (CNS) overgrowth, describe the neuroimaging features and review the underlying genetics. For the organization of this article, we will primarily focus on the major CNS overgrowth syndromes within the PI3K-AKT-mTOR pathway, including *PIK3CA* overgrowth spectrum, *PTEN* hamartoma syndrome and *AKT* related syndromes (Table 1). Additional overgrowth syndromes secondary to other gene mutations will be mentioned briefly at the end.

**Table 1:** Selected syndromes with central nervous system overgrowth

Syndrome	Gene	Clinical features	Main neuroimaging features
CLOVES (*c*ongenital *l*ipomatous *o*vergrowth, *v*ascular anomalies, *e*pidermal nevi, and *s*coliosis/spinal deformities syndrome)	*PIK3CA*	• Thoracic lipomatous overgrowth (distinctive feature) • Segmental and severe somatic overgrowth • Normal head size • Low and high flow vascular malformations, including paraspinal arteriovenous malformations • Epidermal nevi • Scoliosis, sandal gap toes, other musculoskeletal anomalies	• Hemimegalencephaly • Callosal dysgenesis • Scoliosis • Paraspinal arteriovenous malformations, low flow vascular malformations • Lipomatous overgrowth
MCAP (*m*egalencephaly-*c*apillary *m*alformation)	*PIK3CA*	• Macrocephaly • Seizures • Hypotonia • Developmental delay • Capillary malformations that can resemble cutis marmorata • Diffuse somatic overgrowth with mild asymmetry • Syndactyly, sandal gap toes, other musculoskeletal anomalies	• Ventriculomegaly • Megalencephaly or hemimegalencephaly • Cerebellar tonsillar ectopia • Polymicrogyria • Abnormal white matter signal
Cowden	*PTEN*	• Macrocephaly • Various skin findings • Vascular malformations • Developmental delay, including autism • Cancers involving breast, thyroid, endometrial gastrointestinal, renal and melanoma	• Lhermitte-Duclos disease • Increased white matter volume • Enlarged corpus callosum and cerebellum • Ventriculomegaly • Dilated perivascular spaces • Cavernous malformations
Bannayan-Riley-Ruvalcaba	*PTEN*	• Macrocephaly • Intestinal polyposis • Lipomas • Vascular malformations • Pigmented macules of glans penis • Same cancer risk as Cowden syndrome	• Macrocephaly • Dilated perivascular spaces • Excess spinal fat in soft tissues, epidural space and bone marrow
Proteus	*AKT1*	• Usually normal appearance at birth then develops rapidly disfiguring asymmetrical overgrowth • Cerebriform connective tissue nevi • Lipomatous overgrowth and atrophy • Vascular malformations • Thromboembolism • Meningioma, ovarian cystadenoma, parotid adenoma	• Hemimegalancephaly and other malformations of cortical development • Meningioma • Dolichocephaly • Hyperostosis of skull and external auditory canal • Scoliosis
Sotos	*NSD1*	• Dolichocephaly • Frontal bossing • Pointed chin • Developmental delay	• Ventriculomegaly • Cavum abnormalities • Callosal dysgenesis
Weaver	*EZH2*	• Similar to Sotos syndrome • “Stuck on” protruding chin	• Ventriculomegaly • Periventricular leukomalacia • Malformations of cortical development
Beckwith-Wiedemann	11p15.5	• Macroglossia • Hypoglycemia • Visceromegaly • Wilms tumor and other tumors	• Dandy-Walker continuum • Callosal dysgenesis

## Major elements of overgrowth syndromes

**Fig. 1:**
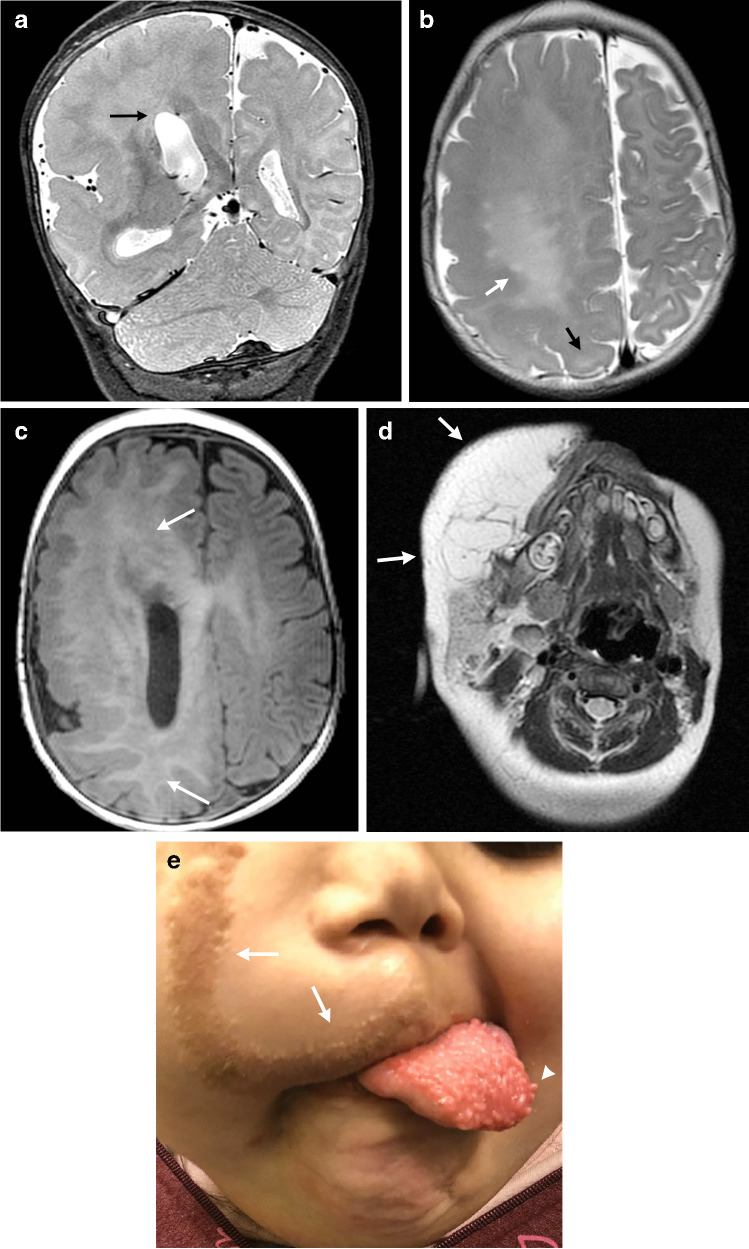
CLOVES (*c*ongenital *l*ipomatous *o*vergrowth, *v*ascular anomalies, *e*pidermal nevi, and *s*coliosis/spinal deformities) syndrome in a 4-month-old girl with a genetically confirmed *PIK3CA* mutation with hemimegalencephaly and facial overgrowth. **a** A coronal T2-weighted image shows right hemimegalencephaly in the cerebrum and cerebellum. The right lateral ventricular body is large and superiorly directed (*arrow*). **b** An axial T2-weighted image shows increased signal within the white matter (*white arrow*). Pachygyria is noted (*black arrow*). **c** An axial spoiled gradient recalled echo image shows increased signal throughout the white matter (*arrows*). **d** An axial T2-weighted image shows substantial lipomatous overgrowth in the right face (*arrows*). **e** A clinical photograph at 5 years of age shows the corresponding right facial overgrowth. Linear epidermal nevi are seen (*arrows*). The tongue is also enlarged with verrucous papules (*arrowhead*). Consent for the photograph was obtained from the patient’s legal guardian

**Fig. 2:**
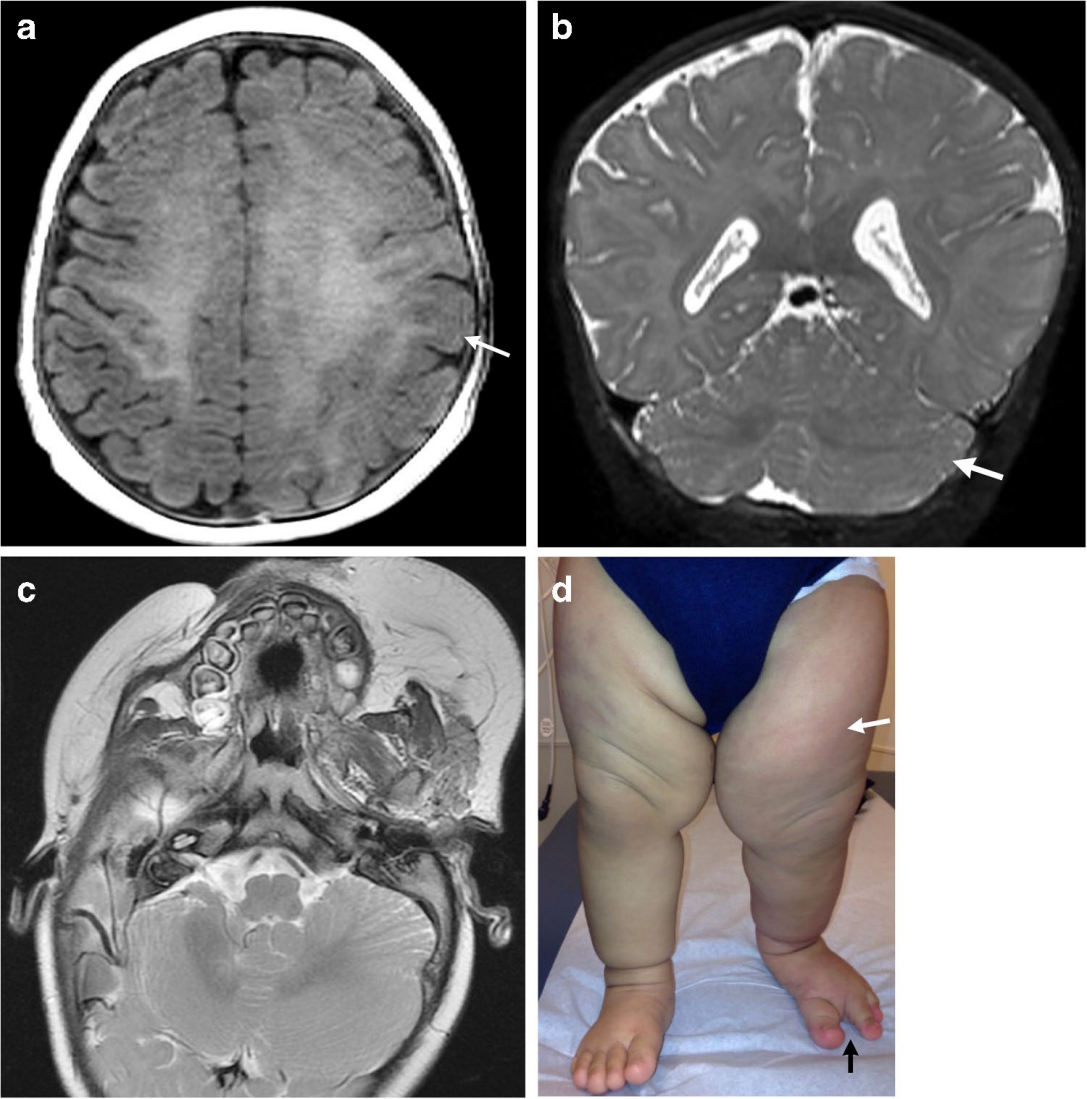
CLOVES syndrome in a 5-month-old boy with genetically confirmed *PIK3CA* mutation with mild hemimegalencephaly and a normal head circumference. **a** An axial spoiled gradient recalled echo image shows mild hemimegalencephaly and subtle pachygyria in the left perirolandic region (*arrow*). **b** A coronal T2-weighted image shows associated enlargement of the left cerebellar hemisphere (*arrow*). **c** An axial T2-weighted image shows lipomatous overgrowth of the left facial soft tissues. **d** A clinical photograph shows subtle overgrowth of the left lower extremity with associated capillary malformations (*white arrow*) and a sandal gap deformity (*black arrow*)

While each of the CNS overgrowth syndromes has unique presentations, main features that may be present include macrocephaly, hemihypertrophy and cancer predisposition. In patients with macrocephaly, hemimegalencephaly is a common manifestation and represents hamartomatous overgrowth secondary to defects in all three phases of cortical development – proliferation, migration and organization. On neuroimaging, hemimegalencephaly shows an enlarged cerebral hemisphere with either a normal cortical surface or pachygyria, polymicrogyria, gray-white blurring, increased T2 signal within the white matter and a characteristic enlarged lateral ventricle with a straight frontal horn (Figs. 1 and 2) [3]. It is important to note that hemimegalencephaly is a dynamic process, changing from a normal size to overgrowth, and can even become atrophic over time.

Hemihypertrophy is secondary to hamartomatous overgrowth in solid organs, bone, muscle, lymphatics and blood vessels. Vascular malformations are common and may either include capillary, venous, lymphatic or arteriovenous malformations. Malignancies can occur in some of the overgrowth syndromes, and cancers may either be within the brain, or more commonly, involving the rest of the organs.

## *PIK3CA*-related overgrowth spectrum (PROS)

*PIK3CA*-related overgrowth spectrum (PROS) is an umbrella term that encompasses a family of genetic syndromes. The *PIK3CA* gene activates the PI3K-AKT-mTOR pathway, which is responsible for cell proliferation, vascular development and angiogenesis [4]. Predominantly somatic gain-of-function mutations in *PIK3CA* result in either isolated anomalies, such as vascular malformations, or result in a syndromic form characterized by overgrowth plus at least two abnormalities in two other systems, including cutaneous/vascular, musculoskeletal, visceral or neurological [5]. These mosaic somatic mutations occur during embryogenesis and result in hamartomatous overgrowth in vessels, lymphatics, muscle, bone and solid organs, which explains why the phenotypes of many PROS syndromes have widespread systemic anomalies. The severity of the phenotypes depends on the level of mosaicism, that is, the balance of the affected and unaffected cells.

Understanding of the PI3K-AKT-mTOR has led to increasing use of targeted drugs to inhibit the pathway and help treat patients afflicted with PROS. Sirolimus is one of the key drugs and functions as an inhibitor of *mTOR* that prevents downstream translation of messenger ribonucleic acid (RNA) and thereby inhibits cellular growth and angiogenesis. In recent years, more targeted therapies of *PIK3CA* now exist, including Alpelisib, which is a *PI3K1* inhibitor. These drugs are now being used in patients with PROS or with extensive vascular anomalies [6].

There are many syndromes within the PROS family, including CLOVES (*c*ongenital *l*ipomatous *o*vergrowth, *v*ascular anomalies, *e*pidermal nevi and *s*coliosis/spinal deformities syndrome), MCAP (*m*egalencephaly-*c*apillary *m*alformation), MPPH (*m*egalencephaly-*p*olymicrogyria-*p*olydactyly-*h*ydrocephalus syndrome), CLAPO (*c*apillary malformation of the lower lip, *l*ymphatic malformation of the face and neck, *a*symmetry and *p*artial/generalized *o*vergrowth), KTS (*K*lippel-*T*renaunay *s*yndrome), FAVA (*f*ibro*a*dipose *v*ascular *a*nomaly) and HHML (*h*emi*h*yperplasia *m*ultiple *l*ipomatosis) [5]. In this review, we will discuss the most common ones with CNS overgrowth that physicians may encounter in clinical practice.

## CLOVES syndrome

### Overview

CLOVES is an acronym referring to congenital lipomatous overgrowth (CLO), vascular anomalies (V), epidermal nevi (E) and skeletal/spinal deformities (S). The syndrome falls under the umbrella PROS. Historically, many patients with CLOVES were previously diagnosed as having Proteus or Klippel-Trenaunay syndrome**,** but with better understanding of the syndromes, correct classification can now be made [4].

### Genetics

Somatic *PIK3CA* gene mutations are the underlying cause of CLOVES syndrome. Molecular diagnosis is best achieved from clinically affected tissue; thereby, DNA sequencing can be performed. Tissue specimens can be obtained via dermal biopsy or surgical excision.

### Clinical manifestations

**Fig. 3:**
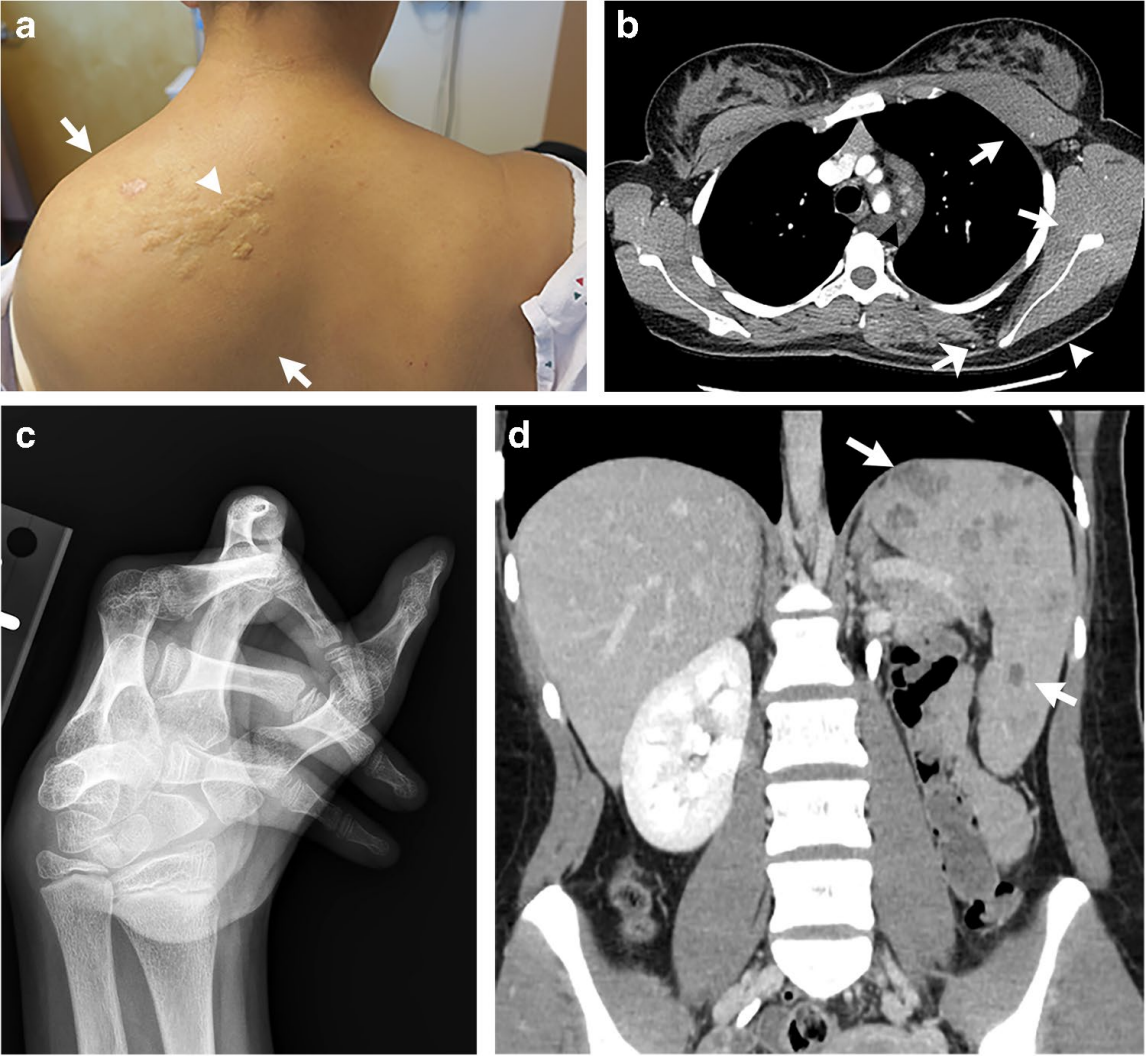
CLOVES syndrome in a 16-year-old girl with a genetically confirmed *PIK3CA* mutation with thoracic lipomatous overgrowth. **a** A clinical photograph shows left upper back overgrowth (*arrows*) that is typical of the CLOVES phenotype. Skin-colored papules (*arrowhead*) were also present that were consistent with collagenomas on biopsy. **b** An axial contrast-enhanced chest computed tomography (CT) shows enlargement of the left anterior and posterior chest wall muscles (*white arrows*) and subcutaneous fat (*white arrowhead*). Venous malformations are present in the left mediastinum (*black arrowhead*) as well as the left back with an associated phlebolith (*wide white arrow*). **c** A posteroanterior radiograph of the hand shows deformity diagnostic of arthrogryposis. **d** A coronal contrast-enhanced CT abdomen shows multiple splenic lesions (*arrows*) that also represent vascular malformations

Thoracic lipomatous overgrowth is one of the most distinctive physical exam features in patients with CLOVES syndrome (Fig. 3). The overgrowth is typically asymmetrical and is usually quite noticeable in many patients. In addition, the overgrowth is congenital in CLOVES syndrome, whereas patients with Proteus syndrome are normal at birth then the overgrowth changes dramatically with age [4]. The head size is usually normal in CLOVES syndrome, while it is large in MCAP [7].

Both high and low flow vascular malformations have been reported in CLOVES syndrome. While high flow malformations are less common, spinal or paraspinal arteriovenous malformations (AVMs) are a characteristic feature [8]. Low flow malformations include lymphatic, venous and capillary malformations (Fig. 3).

Epidermal nevi may be seen and are hamartomatous lesions derived from the epidermis and manifest as hyperpigmented verrucous plaques (Fig. 1). The nevi are usually linear in morphology.

Multiple musculoskeletal abnormalities have been described and include scoliosis, spinal dysraphism, pectus deformities, sandal gap toes (Fig. 2), macro-, poly- and syndactyly, chondromalacia patellae and dislocated knees [5, 8, 9].

### Neuroimaging findings

Neuroimaging features include hemimegalencephaly and agenesis or dysgenesis of the corpus callosum (Figs. 1 and 2). Spinal abnormalities consist of scoliosis, neural tube defects and spinal or paraspinal AVMs with possible ischemic myelopathy. Contrast-enhanced magnetic resonance (MR) angiography can be performed along with traditional spine MR imaging in the setting of an AVM for lesion characterization and potential treatment planning.

Thoracic lipomatous overgrowth can be seen on spine MRI or computed tomography (CT) and is usually over the back or side. Muscular overgrowth can be an accompanying feature as well as cutaneous or deep low flow vascular malformations.

Other than the neuroimaging features, renal agenesis and splenic vascular malformations have been reported (Fig. 3) [5, 9]. Wilms tumor has been reported in a minority of patients with CLOVES syndrome, and some authors recommend abdominal ultrasound screening every three months until the age of 7 [10].

## Megalencephaly-capillary malformation (MCAP) syndrome

### Overview

Megalencephaly-capillary malformation (MCAP) syndrome is characterized by megalencephaly, capillary malformation and somatic overgrowth. The syndrome has been previously referred to as megalencephaly-capillary formation and macrocephaly-cutis marmorata telangiectatica congenita, although MCAP is the name commonly used [11].

### Genetics

The majority of MCAP cases are reported to arise from somatic mosaic mutations in *PIK3CA,* suggesting that the mutation occurred post-fertilization in one cell of the multicellular embryo. For these reasons, genetic analysis from affected tissue is suggested for diagnosis. Germline *PIK3CA* mutations have also been reported from blood sampling but are less common [5, 9].

### Clinical manifestations

The clinical profile in patients with MCAP varies greatly from very mild symptoms to severe. Most patients have enlarged heads due to megalencephaly (occipitofrontal circumference ≥3 standard deviations above the mean) that may either be congenital or present in the early postnatal period and tends to be progressive as the child grows. Because of the abnormal brain growth, developmental delay, seizures and hypotonia are common. Somatic overgrowth may also be present and ranges from facial overgrowth to frank hemihypertrophy. While the aforementioned brain overgrowth is typically progressive, the somatic overgrowth can stabilize over time and even regress. In general, the somatic overgrowth is diffuse with mild asymmetry, whereas the somatic overgrowth in CLOVES patients is typically segmental and severe [7, 11].

**Fig. 4:**
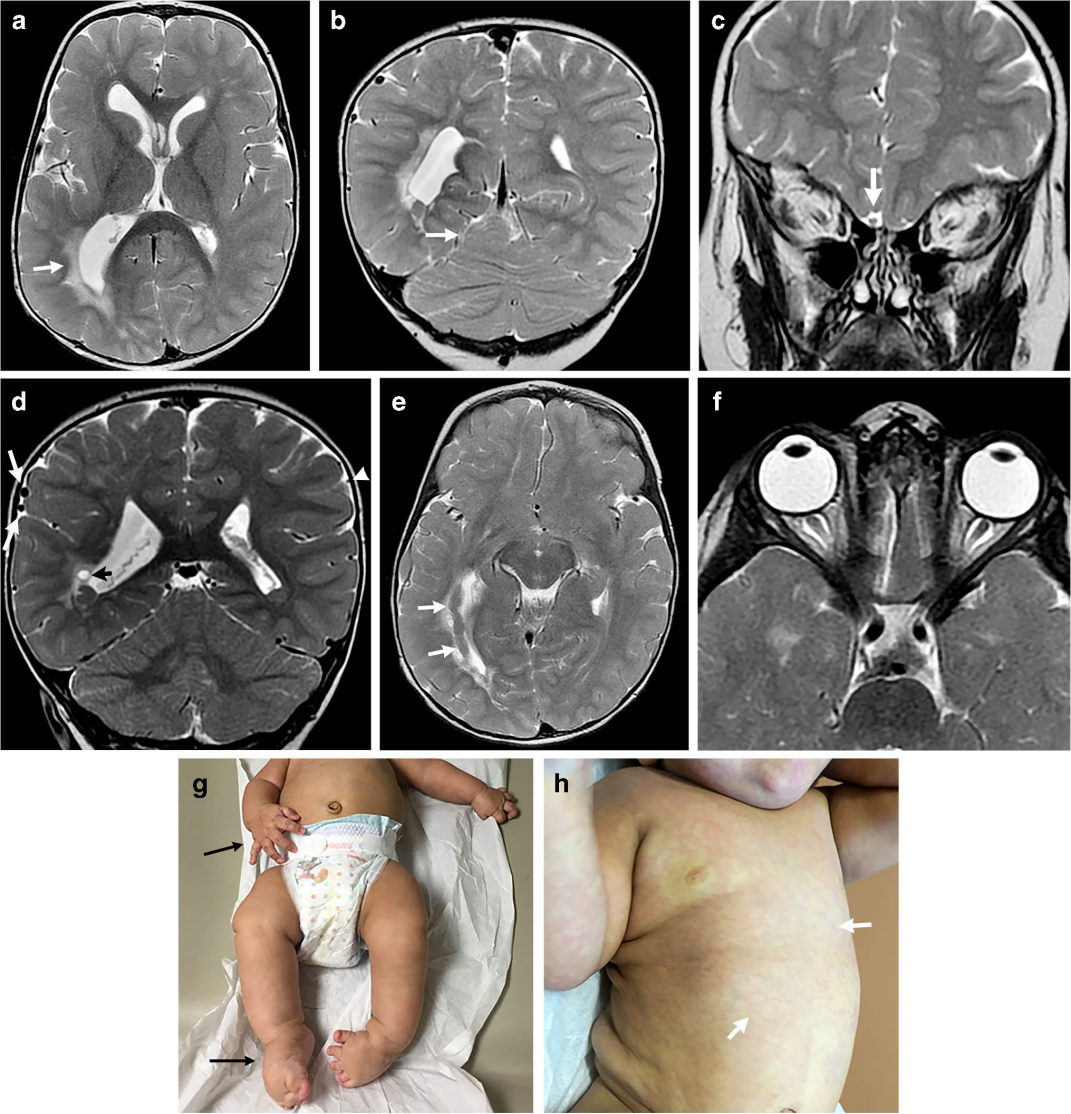
Megalencephaly-Capillary Malformation (MCAP) syndrome in a 19-month-old-boy with a genetically confirmed *PIK3CA* mutation presenting with macrocephaly, facial dysmorphism, somatic overgrowth, capillary malformations, generalized hypotonia and developmental delay. **a-f** Axial and coronal T2-weighted images show an enlarged right cerebral hemisphere compared to the left with associated ventricular enlargement and hyperintense signal within the periventricular white matter (**a**, *white arrow*; **d**, *black arrow*). There is enlargement of the right superior cerebellar vermis (**b**, *arrow*), right olfactory bulb (**c**, *arrow*), and right cerebral cortical veins (**d**, *white arrows*) compared to the normal left cortical veins (**d**, *arrowhead*). Subependymal heterotopia in the right temporal and occipital lobes is present (**e**, *arrows*). Hypertelorism (**f**). **g** A clinical photo shows enlargement of the right upper and lower extremities (*arrows*) with associated deformities and bilateral club feet. **h** A clinical photo shows capillary malformation in a cutis marmorata-like appearance

Capillary malformations are the main vascular anomaly seen in patients with MCAP and can occur in the trunk, limbs and midline face (nevus flammeus). The capillary malformations can be extensive and resemble cutis marmorata (Fig. 4). Infantile hemangiomas and venous malformations have also been reported but are less common [5, 9].

Other physical exam findings include frontal bossing, syndactyly of the 2nd-4th toes or fingers, sandal gap toe deformity, polydactyly, hyperelastic skin and joint laxity. Rarely, cardiac anomalies (atrial and ventricular septal defects and/or abnormalities of the great vessels) and genitourinary abnormalities (cryptorchidism, micropenis, hypospadias, duplicated urinary collection system) may occur [11].

### Neuroimaging findings

Ventriculomegaly or hydrocephalus with or without hemimegalencephaly is the most common neuroimaging finding in patients with MCAP. The etiology is potentially obstructive in some patients with cerebellar overgrowth, but it is unclear if this is the case in all patients [5, 12]. The hydrocephalus can lead to neurosurgical treatment necessitating a ventriculoperitoneal shunt or endoscopic third ventriculostomy [13].

The brain overgrowth in one study of 11 patients with brain MRIs showed 8 with megalencephaly and 3 with hemimegalencephaly [14]. The progressive nature of megalencephaly, particularly if it involves the cerebellum, tends to result in cerebellar tonsillar ectopia because of a crowded posterior fossa [12]. Some studies have reported Chiari I as a feature in MCAP, although it has been suggested that cerebellar tonsillar ectopia is the preferred term given that Chiari I deformities have congenitally small posterior fossas, and in MCAP, the posterior fossa typically becomes crowded due to brain overgrowth [13]. Regardless of the terminology, resultant syringomyelia and suboccipital decompression may be necessary in symptomatic patients.

Polymicrogyria is an increasingly recognized feature with MCAP that is commonly bilateral and perisylvian but may also occur in the bifrontal location or be focal [13]. A thick corpus callosum, abnormal T2 signal in the deep and periventricular white matter (Fig. 4), cavum septum pellucidum or vergae, enlarged dural venous sinuses and prominent perivascular spaces are additional imaging features reported in MCAP [13]. Meningioma and retinoblastoma are rarely reported tumors on neuroimaging [12].

Certain publications recommend surveillance imaging of brain MRIs every 6 months for the first 2 years of life, followed by annually until the age of 8 to monitor for hydrocephalus and cerebellar tonsillar ectopia [5, 9].

MPPH is another overgrowth syndrome that can sometimes mimic MCAP, as there are overlapping imaging features (hemimegalencephaly, polymicrogyria, ventriculomegaly), but intracranial heterotopia, vascular malformations and syndactyly are absent [11].

## *PTEN* hamartoma tumor syndrome

### Overview

Like *PIK3CA* and the PROS family of syndromes, *PTEN* hamartoma tumor syndrome (PHTS) represents a spectrum of disorders secondary to a mutation in the *PTEN* gene. *PTEN* is a tumor suppressor gene and germline mutations result in a loss of function.

The main two syndromes within the PHTS family are Cowden and Bannayan-Riley-Ruvalcaba syndromes, which were initially described as separate disorders but would later be characterized as having a common germline *PTEN* mutation. One of the challenging features in diagnosing patients with PHTS syndrome is the broad phenotypic variability between patients with the same mutation and even with patients in the same family. Major and minor criteria exist for the diagnosis of PHTS but are beyond the scope of this article [15].

### Genetics

Molecular genetic testing can identify heterozygous germline pathogenic variants in the *PTEN* gene.

### Neuroimaging findings

**Fig. 5:**
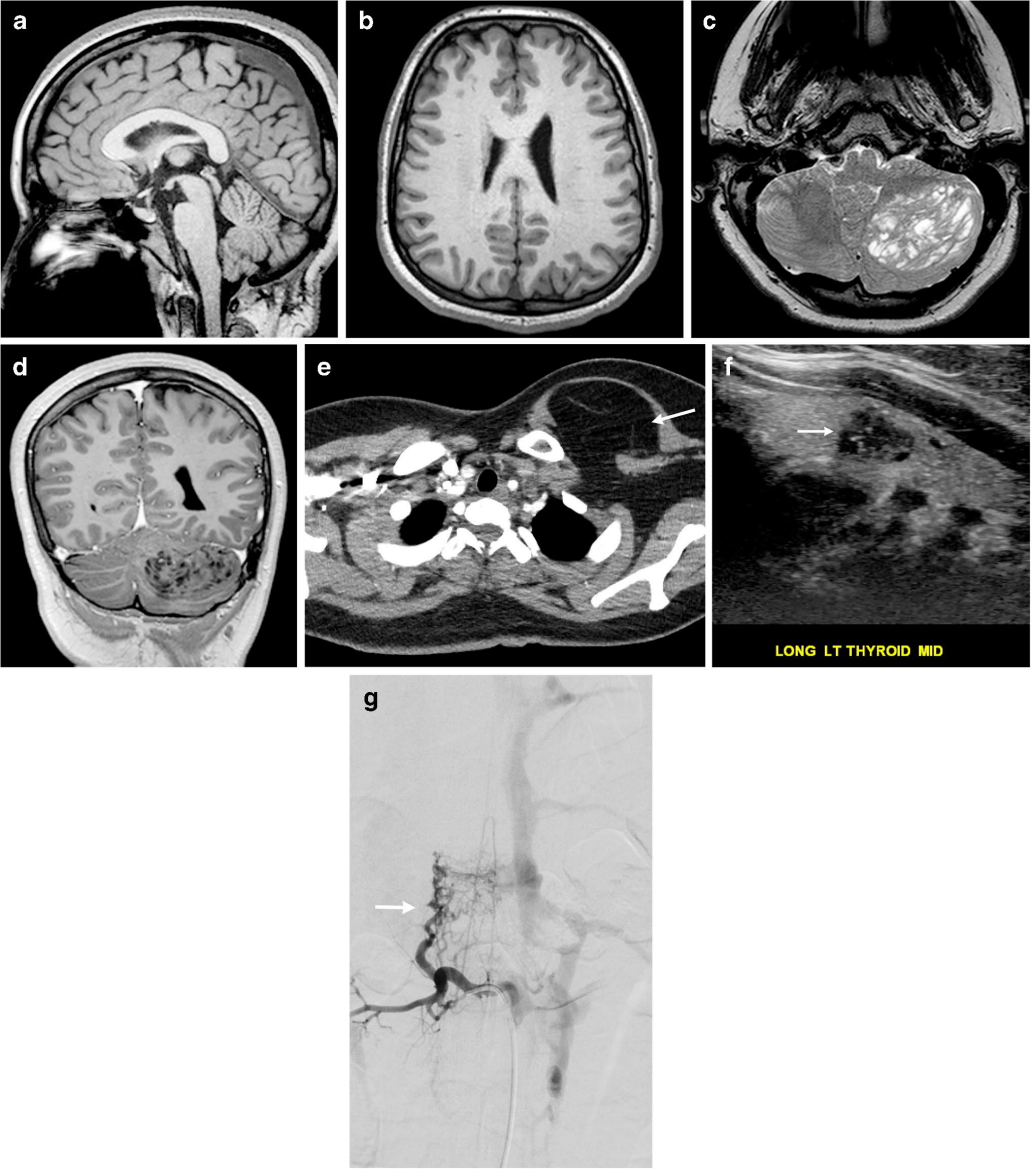
Cowden syndrome in a 14-year-old girl with a genetically confirmed *PTEN* gene mutation with multiple systemic anomalies. **a, b** Sagittal and axial spoiled gradient recalled echo images show a mildly enlarged corpus callosum and excess white matter. **c, d** Axial T2-weighted and coronal contrast-enhanced T1-weighted images show a striated T2 hyperintense nonenhancing lesion in the left cerebellar hemisphere, which was consistent with a dysplastic gangliocytoma—Lhermitte-Duclos disease—after resection. **e** An axial contrast-enhanced chest computed tomography scan shows a large left chest wall lipoma (*arrow*). **f** A longitudinal ultrasound shows a left thyroid nodule (*arrow*) with irregular borders and punctate echogenic foci that was compatible with a papillary thyroid cancer after surgical resection. **g** A conventional spinal angiogram in the anteroposterior projection with the patient supine with injection from the right L1 lumbar artery shows a spinal arteriovenous malformation (*arrow*)

**Fig. 6:**
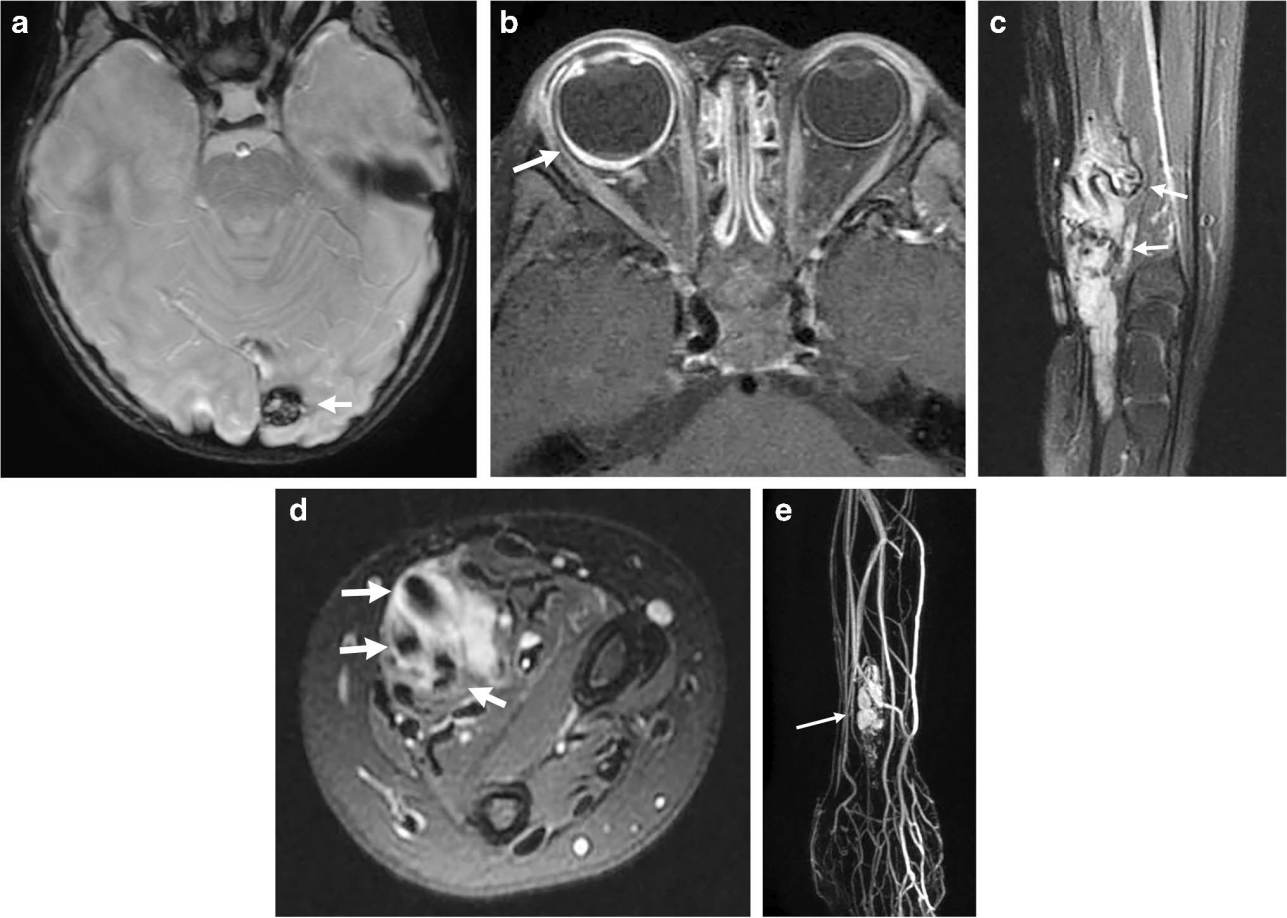
Cowden syndrome in a 3-year-old girl with a genetically confirmed *PTEN* mutation who presented with autism, macrocephaly and a vascular malformation. **a** An axial susceptibility weighted image shows a left occipital cavernous malformation (*arrow*). **b** An axial contrast-enhanced fat-suppressed T1-weighted image of the orbits shows an enlarged right-side globe with abnormal choroidal enhancement (*arrow*) consistent with uveal ganglioneuroma after biopsy and surgical removal. **c, d** Sagittal and axial fat-suppressed T2-weighted images show a predominantly hyperintense intramuscular lesion in the distal forearm and wrist with multiple flow voids (*arrows*). **e** A coronal contrast-enhanced time-resolved magnetic resonance angiogram/magnetic resonance venogram shows arterial enhancement within the lesion (*arrow*), but there are no large draining veins to suggest an arteriovenous malformation. This mass represents a *PTEN* hamartoma of soft-tissue (PHOST) lesion

**Fig. 7:**
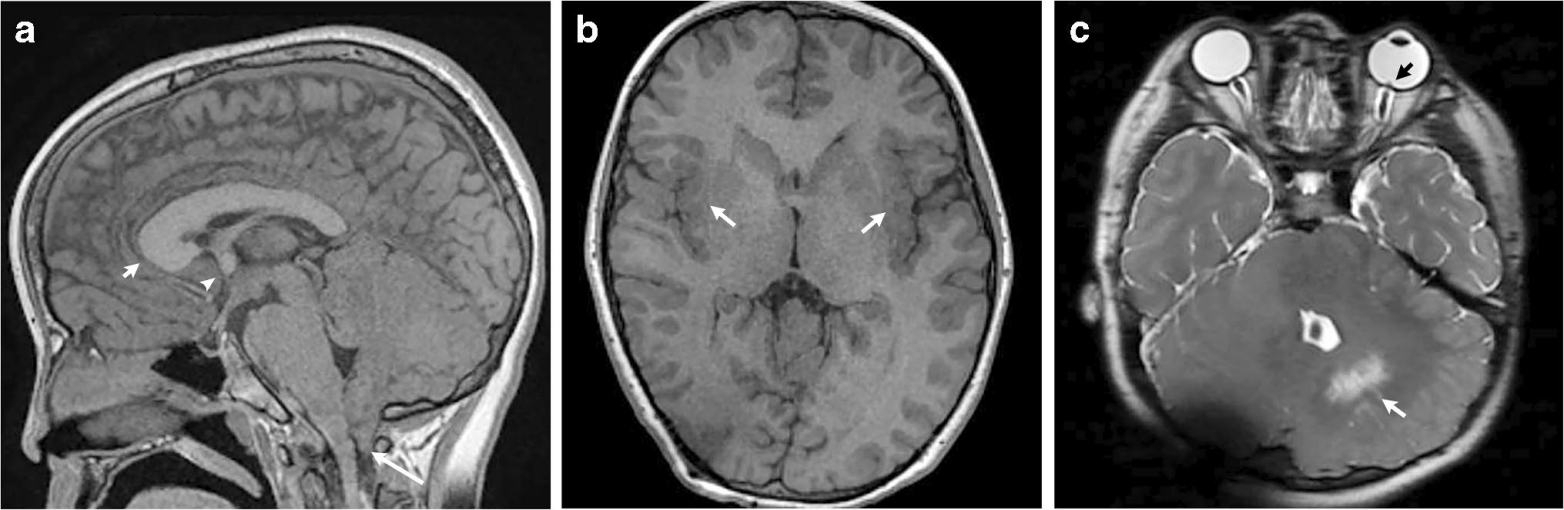
Cowden syndrome in a 10-year-old boy with genetically confirmed *PTEN* mutation. **a** A sagittal spoiled gradient recalled echo (SPGR) image shows an enlarged corpus callosum that is greatest at the anterior aspect (*short arrow*), along with an enlarged anterior commissure (*arrowhead*). The cerebellum is enlarged, and there is tonsillar ectopia (*long arrow*). **b** An axial SPGR image shows bilateral perisylvian polymicrogyria (*arrows*). Note the increased volume of the white matter. **c** An axial T2-weighted image shows marked enlargement of the cerebellar hemispheres with a dysplastic appearance and hyperintense signal on the left (*white arrow*), which likely represents Lhermitte-Duclos disease although it is not classic in appearance. The orbital globes show hypertelorism and papilledema (*black arrow*) from increased intracranial pressure. Susceptibility artifact is noted in the right posterior fossa from a ventriculoperitoneal shunt catheter

While there are unique imaging features within the respective PHTS syndromes, there are common neuroimaging features that can be observed. Increased white matter volume, including an enlarged corpus callosum, is a feature of PHTS and can be assessed qualitatively or with volume segmentation [16]. These findings are one of the principal reasons for macrocephaly in these patients, and hypermyelination is one of the postulated reasons for the increased white matter. PTEN conditional knockout mice have been studied, and brain analysis has revealed enlarged neuronal projections, increased dendritic spine density and increased glial cells, leading to the hypermyelination [16]. Enlargement of the cerebellum and lateral ventricles can be another cause of macrocephaly but is less severe compared to the white matter overgrowth [17]. Lhermitte-Duclos disease can be observed and is a benign lesion representing a dysplastic gangliocytoma or hamartoma. This entity always occurs in the cerebellum and shows T2 hyperintense white matter signal with a normal cortex in a striated or tigroid pattern. Facilitated diffusion and no or mild enhancement are observed after contrast administration [18]. Additional imaging features in PHTS patients include multiple dilated perivascular spaces, white matter signal abnormalities, decreased cortical thickness, vascular malformations such as cavernous malformations, developmental venous anomalies or dural arteriovenous fistulas, cortical malformations and hemimegalencephaly, and cerebellar ectopia (Figs. 5, 6 and 7) [17, 19, 20].

In addition to routine vascular malformations that can occur throughout the body, patients with PHTS can exhibit a unique vascular malformation called PTEN hamartoma of soft tissue (PHOST) (Fig. 6). These lesions are usually complex-appearing and can demonstrate slow and high flow components. Fibrofatty infiltration of muscle, fascia and even bone may be seen. Because of the diverse features within PHOST lesions, these masses are often misdiagnosed in the absence of a known *PTEN* mutation. Differential diagnoses can include venous malformation, arteriovenous malformation and fibroadipose vascular anomaly [21].

## Cowden syndrome

### Overview

Cowden syndrome is a rare multisystemic disease characterized by overgrowth of various tissues and increased risk for several malignancies throughout the body. Most patients present in early adulthood with macrocephaly, characteristic skin lesions, hamartomas and malignancies.

### Genetics

Cowden syndrome is autosomal dominant and is caused by inherited or de novo mutations in *PTEN*.

### Clinical manifestations

More than 90% of patients with Cowden syndrome have clinical manifestations in their late 2nd decade, and by the fourth decade, 99% have some clinical features [22]. On physical exam, patients usually have macrocephaly and dolichocephaly. A skin exam can show trichilemmomas (benign follicular epithelial neoplasms), papillomatous papules (skin-colored raised bumps), acral and plantar keratoses (dark flat spots on hands and feet) and vascular malformations. Autism spectrum disorder and developmental delay may be observed [22].

Patients with Cowden syndrome have a very high risk of developing malignancies throughout the body, including breast, thyroid, endometrial, gastrointestinal, renal and cutaneous melanoma. Cancer screening guidelines exist for each of these malignancies to ensure early detection.

### Neuroimaging findings

Neuroimaging in patients with Cowden syndrome classically shows Lhermitte-Duclos disease. In addition, meningiomas have been reported with Cowden syndrome, but it is unclear if there is a true higher incidence, as meningiomas are common in the general population.

## Bannayan-Riley-Ruvalcaba syndrome

### Overview

This syndrome classically exhibits the cardinal features of macrocephaly, hamartomatous intestinal polyposis, lipomas, vascular malformations and pigmented macules of the glans penis.

### Genetics

Bannayan-Riley-Ruvalcaba syndrome is an autosomal dominant disorder, and *PTEN* mutations have been reported in 60% of patients.

### Clinical manifestations

In addition to the classic triad of macrocephaly, lipomatosis and pigmented macules of the glans penis, these patients can have high birth weight, myopathies in proximal muscles, joint hyperextensibility, pectus excavatum, scoliosis and abnormal facial features [22]. Developmental and motor delays are observed, and most patients are diagnosed in childhood. Formal diagnostic criteria have not been agreed upon, but diagnosis is established based on the combination of genetic analysis, clinical and radiologic features. Cancer risk and screening of breast, thyroid, endometrial, gastrointestinal, renal and cutaneous melanoma are similar to Cowden syndrome, although no formal studies have studied malignancy risk in this patient population [22].

### Neuroimaging findings

**Fig. 8:**
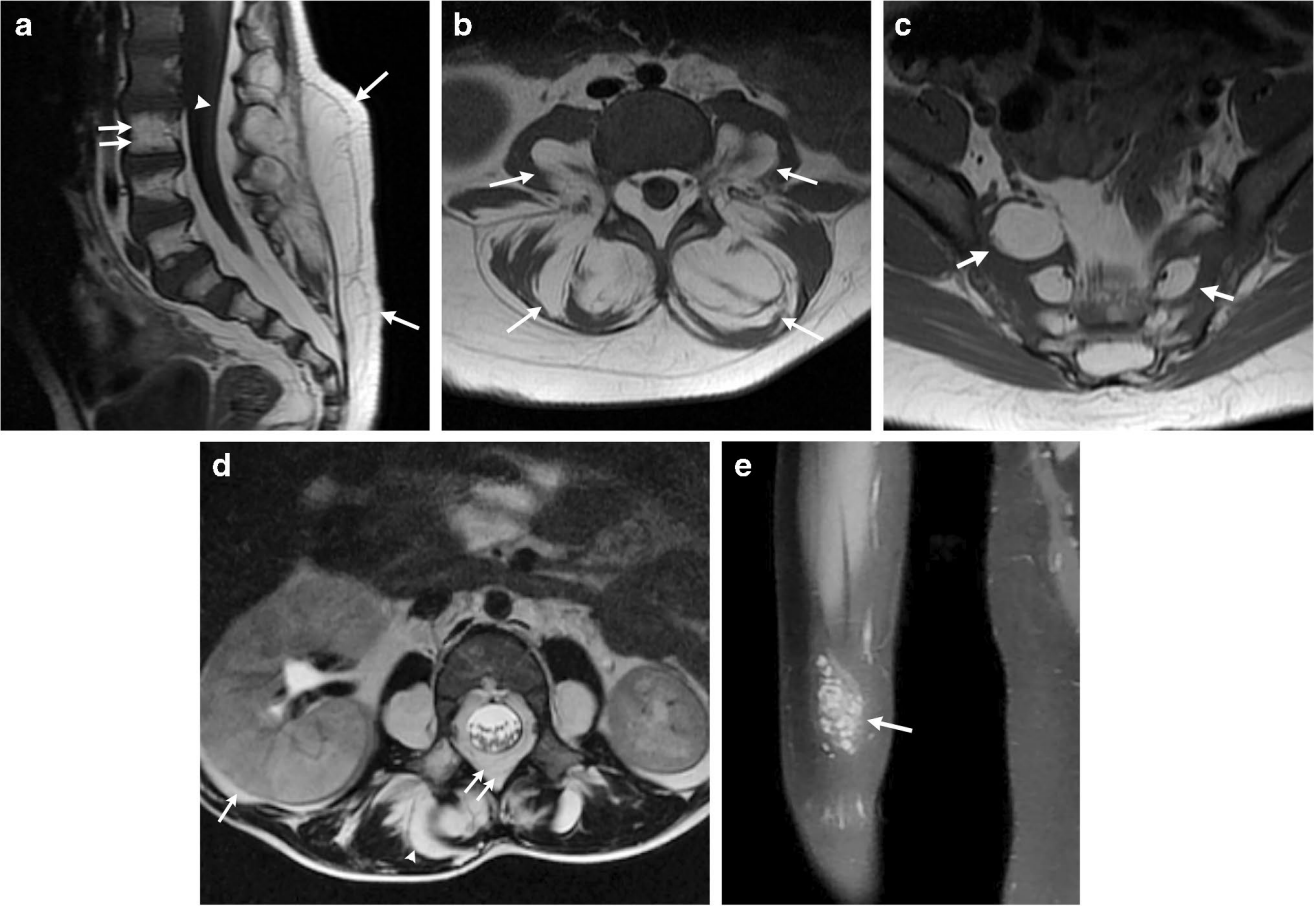
Bannayan-Riley-Ruvalcaba syndrome in a 3-year-old boy with a genetically confirmed *PTEN* mutation with multiple anomalies. **a-c** Sagittal and axial T1-weighted images show excessive fat throughout the lumbosacral spine. In (**a**), there is cutaneous and subcutaneous dorsal lipomatous overgrowth (*arrows*), extensive dorsal and ventral epidural fat (*arrowhead*) and abnormal fat within the vertebral body bone marrow (*double arrows*). In (**b, c**), extensive fatty replacement is observed within the psoas and paraspinal muscles (*arrows* in **b**) and along the sacral neural foramina (*arrows* in **c**). **d** An axial T2-weighted image shows right-side nephromegaly (*arrow*) as well as the excess fat in the paraspinal tissues (*arrowhead*) and epidural fat (*double arrows*). **e** A coronal contrast-enhanced T1-weighted image shows a solid enhancing mass in the ventral forearm consistent with a venous malformation

Macrocephaly and dilated perivascular spaces have been reported on patients with Bannayan-Riley-Ruvalcaba syndrome [23]. Spinal anomalies have also been reported in these patients, and the main feature is excess fat. On imaging, patients can exhibit lipomatous overgrowth in the subcutaneous tissues, epidural lipomatosis, fat within the muscles and neural foramina, and even fatty conversion of bone marrow (Fig. 8) [23]. The lipomatosis can be progressive.

## Proteus syndrome and AKT anomalies

### Overview

Proteus syndrome is an overgrowth syndrome that results in severe overgrowth and disfigurement. The disease is exceptionally rare, with a very rough prevalence estimate of 1 out of 1–10 million people [24]. Furthermore, some of the previous reports in the literature were published before modern genomic testing, and some of these reports may have represented *PIK3CA* or other mutations rather than represent Proteus syndrome [25].

### Genetics

Proteus syndrome is caused by a somatic activation mutation in *AKT1*, which is responsible for cell growth and proliferation [26]. Tissue sampling from affected tissue can detect *AKT1*.

### Clinical manifestations

**Fig. 9:**
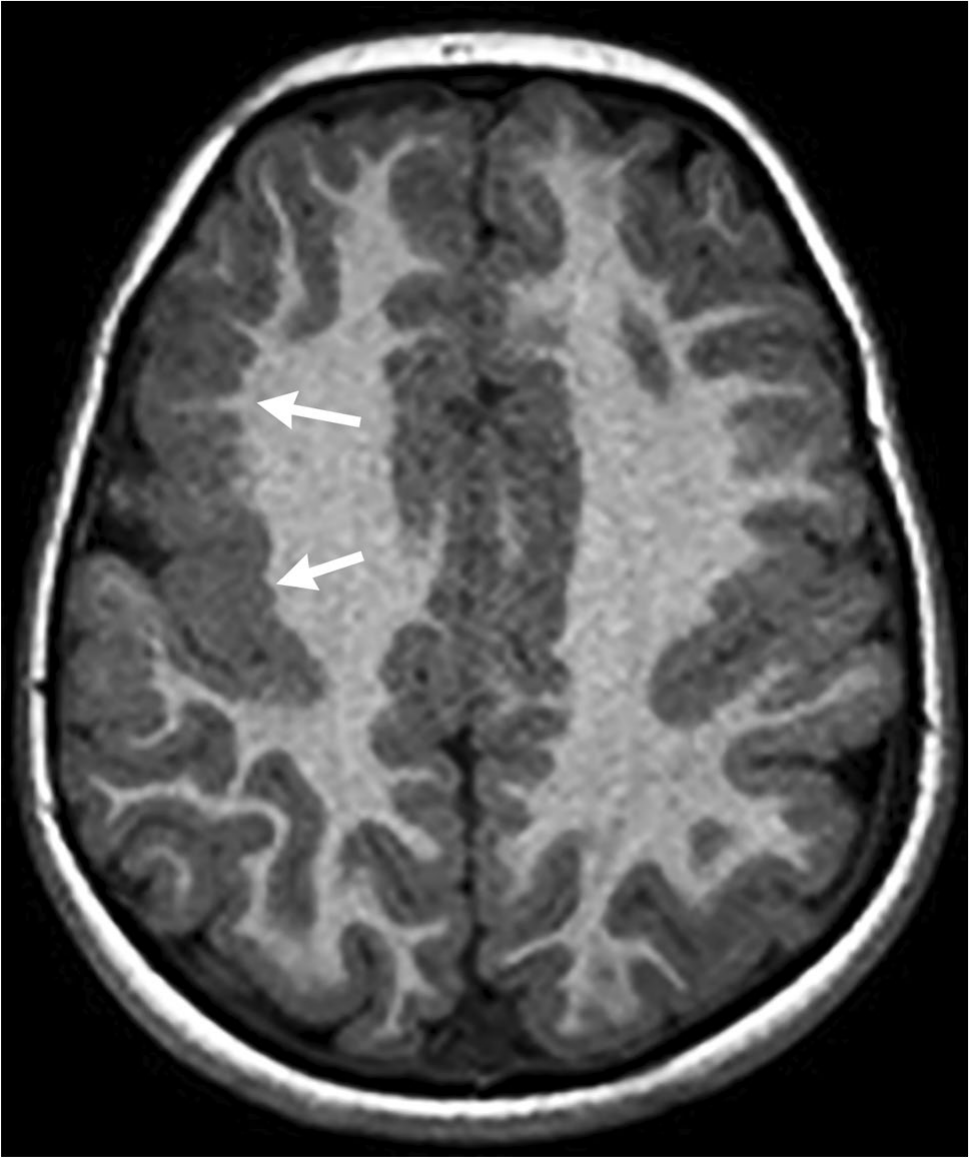
*AKT3* genetically confirmed mutation. An axial spoiled gradient recalled echo image shows extensive polymicrogyria within the right frontal lobe (*arrows*). Image courtesy of A.J. Barkovich, MD

The growth pattern of Proteus syndrome is unique and helpful in making the diagnosis. Patients have little or no manifestations at birth but rapidly develop disfiguring asymmetrical and disproportionate skeletal overgrowth in childhood. This pattern separates it from other overgrowth syndromes, especially *PIK3CA*. While the somatic overgrowth is usually progressive, macrocephaly secondary to hemimegalencephaly can be congenital. To date, no *AKT1* mutations have been identified in affected brain tissue, and this is likely due to its inherent low levels of brain expression. *AKT3* mutations, on the other hand, have higher levels of brain expression and readily result in malformations of cortical development (Fig. 9), although this gene does not result in Proteus syndrome [27, 28].

Cerebriform connective tissue nevi is essentially pathognomonic for Proteus syndrome and are nevi that resemble the brain gyration pattern. They commonly occur on the hands and feet, but other locations have been reported. Linear verrucous epidermal nevi are another dermatologic finding. Patients often have dysmorphic facial features that develop in childhood [26].

Patients often develop alterations in fat and can both develop lipomatous overgrowth and atrophy. Vascular malformations have been reported, including capillary, lymphatic and venous subtypes, while arteriovenous malformations are rare. Thromboembolism in the deep veins and pulmonary arteries is a potential risk. There is an increased predisposition of certain tumors, including meningioma, ovarian cystadenoma and parotid adenoma. Psychosocial issues and developmental delay may be witnessed, although the majority of patients have normal intelligence [29].

### Neuroimaging findings

Hemimegalancephaly and other malformations of cortical development can be observed in patients with Proteus syndrome. Meningiomas, dolichocephaly and hyperostosis of the skull and external auditory canal have been reported. Within the spine, the vertebral bodies can abnormally enlarge and result in scoliosis [24].

## Additional syndromes not related to PI3K-AKT-mTOR pathway

### Sotos syndrome

#### Overview

Sotos syndrome is an overgrowth syndrome typified by the cardinal features of a distinctive facial appearance, developmental delay and childhood overgrowth. Cerebral gigantism was used historically as another name of the syndrome.

#### Genetics

The syndrome is caused by mutations in the *NSD1* gene, which is responsible for normal embryonal development. While the syndrome can be transmitted in an autosomal dominant manner, most cases are de novo germline mutations [30].

#### Clinical manifestations

Patients with Sotos syndrome usually manifest with characteristic facial features of a dolichocephaly, frontal bossing, minimal hair in the frontotemporal scalp, malar flushing, down-slanting palpebral fissures and a pointed chin. There is overgrowth in height and/or head circumference. Seizures, scoliosis, hearing loss, advanced bone age and joint laxity may be accompanying features. Patients usually have delayed language and motor development [30, 31]. Acute myelocytic leukemia has been reported in a small percentage of patients [32].

#### Neuroimaging findings

**Fig. 10:**
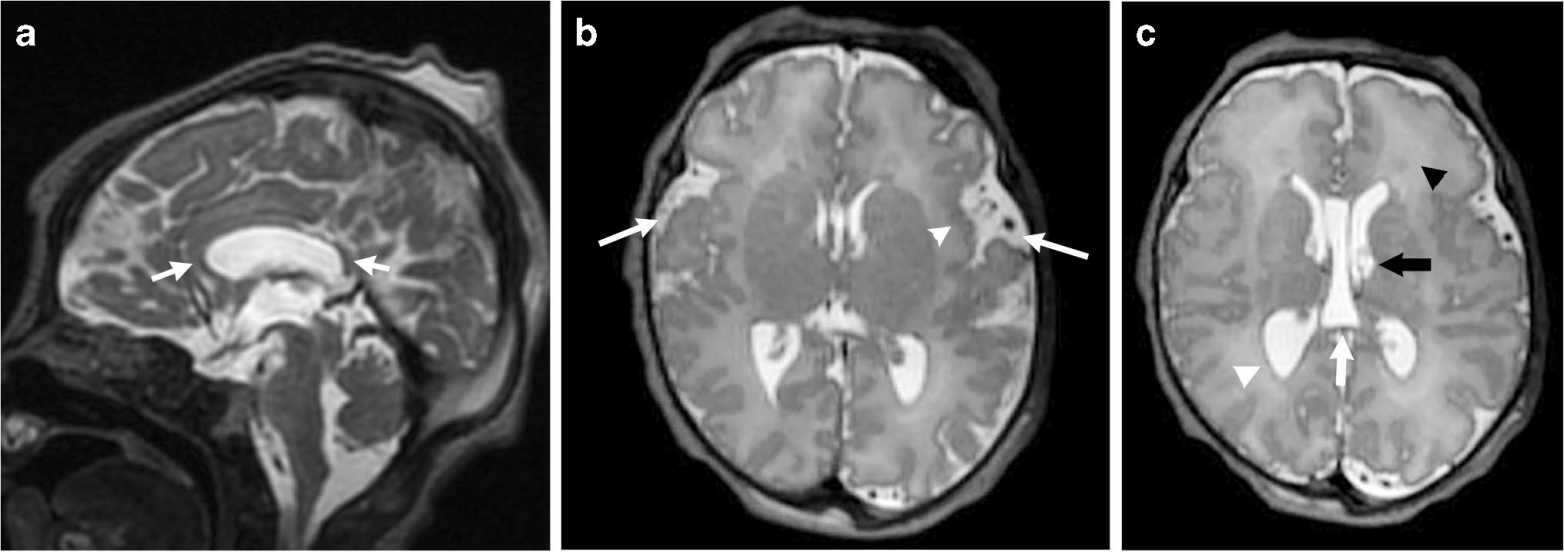
Sotos syndrome in a 4-day-old boy with genetically confirmed *NSD1* gene mutation. **a** A sagittal T2-weighted image shows a small corpus callosum (*arrows*). Scalp swelling is noted from recent delivery. **b** An axial T2-weighted image shows widened frontal opercula (*arrows*) and left perisylvian polymicrogyria (*arrowhead*). **c** An axial T2-weighted image shows ventriculomegaly with large trigones (*white arrowhead*), cavum septum pellucidum and vergae (*white arrow*), and diffusely increased T2 signal within the white matter (*black arrowhead*). Periventricular cysts are also noted (*black arrow*) that could be related to the syndrome or be sequala from previous germinal matrix hemorrhages

**Fig. 11:**
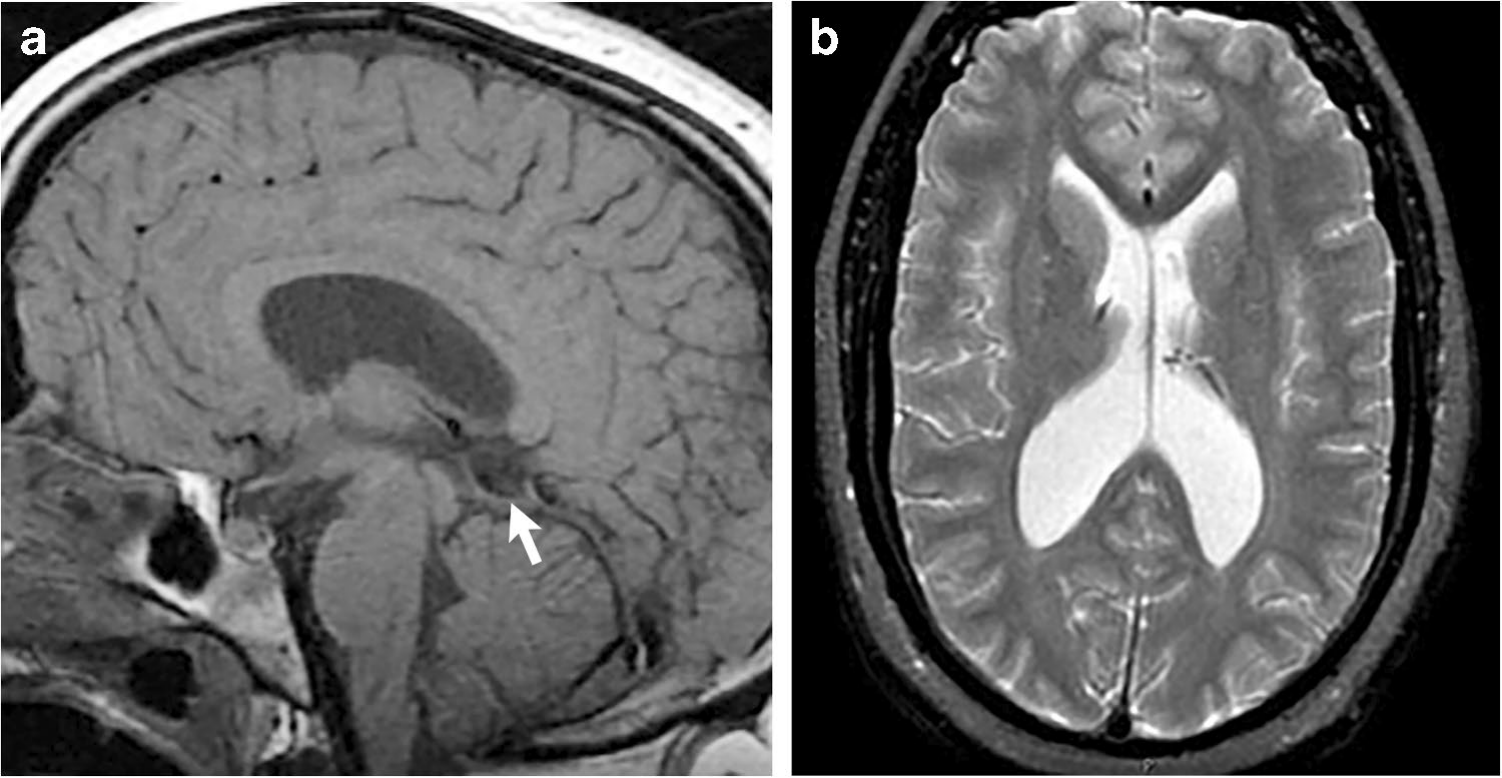
Sotos syndrome in a 17-year-old-girl with developmental delay. **a** A sagittal T1-weighted image shows a midline cyst (*arrow*) superior to the cerebellar vermis. **b** An axial T2-weighted image shows ventriculomegaly with large trigones

The neuroimaging features in Sotos syndrome mostly involve the ventricular system. These include ventriculomegaly, prominent trigones and cavum abnormalities (cavum septum pellucidum, verage or velum interpositum). Callosal dysgenesis has also been reported, along with periventricular signal abnormalities, subependymal cysts, widen opercula, enlarged subarachnoid spaces, gray matter heterotopias and enlarged cerebellum (Figs. 10 and 11) [32, 33].

### Weaver syndrome

Weaver syndrome is secondary to an *EZH2* mutation, which is responsible for normal embryonal development. The syndrome shares some clinical features similar to Sotos syndrome but with a “stuck on” protruding chin that may be one of the differentiating features on physical exam. Neuroimaging has only been reported in a few patients, but ventriculomegaly, periventricular leukomalacia and malformations of cortical development have been reported [34].

### Beckwith-Wiedemann syndrome

Beckwith-Wiedemann syndrome is an overgrowth disorder secondary to epigenetic alterations in growth regulatory genes at 11p15.5, primarily due to genetic imprinting. Cardinal features of this common overgrowth syndrome consist of macroglossia, persistent hypoglycemia, visceromegaly and Wilms tumor. Intracranial anomalies have been reported in a minority of patients and consist of malformations within the Dandy-Walker continuum as well as callosal dysgenesis [35].

## Conclusion

There are many overgrowth syndromes with central nervous system involvement that usually manifest with other systemic findings in the body. The radiologist should incorporate all elements of the clinical history, physical exam, genetics and radiology of other body parts during image interpretation. In this pictorial essay, we have discussed the main overgrowth syndromes with brain and spine involvement, highlighted key neuroimaging features and reviewed the underlying genetics.
